# Single-institution cross-sectional study to evaluate need for information and need for referral to psychooncology care in association with depression in brain tumor patients and their family caregivers

**DOI:** 10.1186/s40359-020-00460-y

**Published:** 2020-09-10

**Authors:** Christiane Reinert, Michael Gerken, Katharina Rathberger, Katharina Krueger, Monika Klinkhammer-Schalke, Patricia Lindberg-Scharf, Oliver Koelbl, Martin A. Proescholdt, Markus J. Riemenschneider, Tobias Pukrop, Elisabeth Bumes, Markus Hutterer, Peter Hau

**Affiliations:** 1 Department of Neurology and Wilhelm Sander-NeuroOncology Unit, University Hospital Regensburg, Franz-Josef-Strauß-Allee 11, Universitätsstrasse 84, 93053 Regensburg, Germany; 2 Tumor Center - Institute for Quality Assurance and Health Services Research, University of Regensburg, Am Biopark 9, 93053 Regensburg, Germany; 3 Department of Radiotherapy and Radiation Oncology, University Hospital Regensburg, Franz-Josef-Strauß-Allee 11, 93053 Regensburg, Germany; 4 Department of Neurosurgery, University Hospital Regensburg, Franz-Josef-Strauß-Allee 11, 93053 Regensburg, Germany; 5 Department of Neuropathology, Regensburg University Hospital, Franz-Josef-Strauß-Allee 11, 93053 Regensburg, Germany; 6 Department of Internal Medicine III, University Hospital Regensburg, Franz-Josef-Strauß-Allee 11, 93053 Regensburg, Germany; 7 Department of Neurology 1, NeuroMed Campus, Kepler University Hospital Linz, Wagner-Jauregg-Weg 15, A-4020 Linz, Austria

**Keywords:** Brain tumor, Patient, Family caregiver, Psychooncologic need, Need for referral to psychooncology care, Information need, Depression

## Abstract

**Background:**

The prognosis of patients with brain tumors is widely varying. Psychooncologic need and depression are high among these patients and their family caregivers. However, the need for counselling and need for referral to psychooncology care is often underestimated.

**Methods:**

We performed a single-institution cross-sectional study to evaluate psychooncologic need, depression and information need in both patients and their family caregivers. The Hornheider Screening Instrument (HSI) and the Patient Health Questionnaire (PHQ-9) were used to evaluate psychooncologic need and depression, and a study-specific questionnaire was developed to evaluate information need. Multivariable analyses were performed to detect correlations.

**Results:**

A total of 444 patients and their family caregivers were approached to participate, with a survey completion rate of 35.4%. More than half of the patients and family caregivers were in need for referral to psychooncology care and 31.9% of patients suffered from clinically relevant depression. In multivariable analysis, psychooncologic need were positively associated with mild (odds ratio, OR, 7.077; 95% confidence interval, CI, 2.263–22.137; *p* = 0.001) or moderate to severe (OR 149.27, 95% CI 26.690–737.20; *p* <  0.001) depression. Patient information need was associated with depression (OR 3.007, 95% CI 1.175–7.695; *p* = 0.022).

**Conclusions:**

Unmet counselling need in brain tumor patients and their family caregivers associate to high psychooncologic need and depression. Adequate information may decrease the need for referral to psychooncology care and treatment of depression in these patients. Future studies should further explore these relations to promote development of supportive structures.

## Background

Primary tumors of the central nervous system (CNS) have an average annual age-adjusted incidence of 5.47 per 100,000 population [1]. Brain tumors are a heterogeneous group of diseases that are associated with widely varying prognoses.

Brain tumor patients are a specific population as they often suffer from neurocognitive problems and depression, that may influence need for referral to psychooncology care and information need. A wealth of publications and clinical experience demonstrates that the requirement for patient-oriented information, counselling, and need for referral to psychooncology care is high in these patients [2, 3]. Furthermore, needs and preferences are heterogenous amongst patients and caregivers [4].

Recent publications show that more than half of patients with glioma report distress during their disease course, which partly resolves after active treatment is finished. However, subjects with high distress at diagnosis remain highly distressed at follow-up [3]. Younger age, functional impairment, and concurrent major depression are independently associated with high distress levels. The most frequently reported causes of distress are worry, fatigue, sleep difficulties, and sadness [5].

The supportive needs of brain tumor patients are high and depend on distress and other patient- and disease-related factors [6–8]. Family caregivers - who also have high supportive care requirements which persist over time - should be included in the supportive setting [9, 10]. Adequate information is one important component of a supportive structure [11]. Provision of individual information can reduce distress [12] and help patients to cope with their disease [13, 14]. The quality of life of patients and caregivers depends on coping strategies, which, in turn, are based on support [15].

Previous studies have shown that at least 25–30% of all cancer patients require referral to psychooncology care. This is particularly true for patients with a poor prognosis [16, 17]. Referral to psychooncologic care has positive effects in terms of the burden of diagnosis and coping with the disease [18], and may also prolong survival time [19].

Young adults may have different communication and care preferences to older persons [20]. A greater unmet information need is associated with lower overall mental and physical quality of life. In the younger population, almost half of patients report a negative impact of cancer on personal self-control. In this subpopulation, perceived control and unmet information need were independently associated with quality of life [21].

In clinical routine, however, the need for information and need for referral to psychooncology care is often underestimated [22]. In addition, it has not yet been investigated how adequate, patient-centered measures can best be provided for brain tumor patients and their family caregivers.

The aim of this cross-sectional study was to evaluate the relationships between tumor burden and information need in patients with brain tumors and their family caregivers. Using multivariable analyses, known prognostic factors and other potential cofounders were evaluated to detect correlations between psychooncologic need, information need, and depression in patients and family caregivers.

## Methods

### Patients and relatives

This single-center cross-sectional study included all adult and legally competent patients with a World Health Organization (WHO) grade I to IV brain tumor who were treated at the Regensburg Brain Tumor Centre between January 2014 and September 2015. Histologies included astrocytoma, oligoastrocytoma, oligodendroglioma, glioblastoma, ependymoma, primary central nervous system (CNS) lymphoma, and meningioma. Patients and their family caregivers were questioned once at a specific cut off-date (questionnaires mailed 01.09.2015). Patients were undergoing first-line or recurrence treatment with various modalities (surgery, radiotherapy, systemic therapy, experimental therapy) at the time the questionnaire was completed.

The mailed package included one set of questionnaires for the patient and a second set to be passed on to a family caregiver/relative. Patients and caregivers both received the Hornheider Screening Instrument (HSI), Patient Health Questionnaire (PHQ-9) and an in-house brain tumor-specific self-assessment questionnaire. No reminder was sent. All questionnaires returned by the end of December 2015 were evaluated. Patients and family caregivers decided whether their data would be stored pseudonymized or in identifiable form. If storage of personalized data was selected, patient information materials were sent or referral to psychooncology care was offered as required on the basis of the returned questionnaire.

Participation was voluntary, and patients and family caregivers could decline to participate. The local ethics committee of the University of Regensburg issued a positive vote on the conduct of this study (number 14–101-0291). All procedures were performed according to the ethical standards laid down in the 1964 Declaration of Helsinki and its later amendments. This non-interventional investigation was not registered in a publicity accessible database.

### Questionnaires

We used the Hornheider Screening Instrument (HSI), an established screening tool to sort patients into groups with and without need for referral to psychooncology care, to evaluate psychooncologic need [23]. The data of the patients questionnaires were analyzed via summation of the item scores to a summed score ranging from 0 to 14, with higher scores indicating an increased psychooncologic need. We defined that a patient required referral to psychooncology care if the summed score was > 3, and thereby constructed two groups for further evaluation, with a score of 0–3 and 4–14 [24].

The Patient Health Questionnaire (PHQ-9), a published and broadly used instrument, was used to assess depression [25]. PHQ-9 diagnoses a likely major depression if five or more symptoms of depression occurred on more than half the days of the prior week, and if at least one cardinal symptom is present. The summed score correlates to the extent of depression. In this study, we graded 0–4 points as no depression, 5–9 as mild depression, 10–14 as moderate depression, and ≥ 15 points as severe depression and categorized patients into the three groups 0–4, 5–9 and above 10 for further analysis. Values greater or equal to 10 were classified as clinically relevant [26].

Based on frequently asked questions, an in-house brain tumor-specific self-assessment questionnaire, which is available in German language, was developed to evaluate the information approach, information behavior, information level, and information requirements of patients and family caregivers. The questions were tested and adapted in a construction cohort [27] and were later extended to the current population. In the first part of the questionnaire, medical and sociodemographic data such as sex, age, education level, working situation, brain tumor diagnosis, treatment situation, and approach to psychooncology care and social care were collected in a standardized manner. In the second part, participants were asked to describe how and to what extent they wished to be informed about their disease and its treatment. The third part covered five areas of questions on information need (specific question: “On which topics can we currently support you with information?”) concerning diagnosis, treatment, living with cancer, additional support, and legal issues, which included brain tumor-specific items. If at least in one of the five topics a support with information was desired, we categorized the patient or caregiver as having information need.

All questionnaires were used in the mother language of patients and family caregivers, German.

### Hypotheses, aims and comparison of groups

Our main hypothesis was that psychooncologic need and depression burden may associate with counselling and information need.

Our primary aim was to identify relationships between psychooncologic need, information need, and depression in patients with primary brain tumors and their caregivers. Secondary aims were to describe the items listed above, to inform counselling of future patients, and to generate hypotheses for further research.

### Statistics

For continuous data, descriptive statistics were applied (SPSS version 25.0; IBM Corp., Armonk, NY, USA) using the mean, median, minimum, maximum, and standard deviation. Categorical data are presented as absolute and relative frequencies. Continuous variables were compared between patients and family caregivers using the *t*-test for normally distributed data and the Mann–Whitney U test for non-normally distributed data. The independence of categorical variables was tested with Pearson’s chi-squared test at a significance level of 5%. In case of small numbers, the Fisher’s exact test was applied.

A multivariable binary logistic regression analysis was performed to control for known confounding factors. Known prognostic factors as age, sex, marital status, education level, working situation, WHO grade (grade I or II vs. III vs. IV) [28], tumor status (primary or relapse), time from diagnosis, and treatment status (surgery and radiotherapy vs. chemotherapy vs. follow-up) were corrected for in multivariable analysis. Results were presented as odds ratios (OR) with 95% confidence intervals (95% CI).

Potential sources of bias were addressed by approaching a consecutive series of patients with predefined characteristics, by approaching them without preselection, by processing all available data in a pseudonymized way, and by using statistical methods that are suited to minimize bias.

## Results

### Patients and family caregivers

In total, 444 patients were approached to fill in the questionnaire. For each patient, a questionnaire for a family caregiver was added to the mailed package, which could be delivered to any relative within the family. Of the 444 patients approached, 172 (38.7%) took part in the survey. Within the participating patient cohort, 142 family caregivers (82.6%) also responded. Details are shown in the CONSORT diagram (Additional file 1 [27];). Patient and family caregiver characteristics are outlined in Table 1.

**Table 1:** Characteristics of patients and family caregivers. Distribution of relevant patient characteristics. Significant differences are shown in bold

**Variable**	**Code**	**Patients** ***N*** **= 172**		**Family caregivers** ***N*** **= 142**		**Chi-square** **(*p*-value)**
**Sex**	Male	75	43.6%	66	46.5%	0.446
	Female	97	56.4%	74	52.1%	
	Unknown	0	0%	2	1.4%	
**Age (mean)**		51.4 years [min 20 / max 84]		53.5 years [min 19 / max 82]		–
**Age categories (years)**	≤35	31	18.0%	14	9.9%	0.107
	36–50	45	26.2%	39	27.5%	
	51–65	68	39.5%	61	43.0%	
	> 65	28	16.3%	25	17.6%	
	Unknown	0	0%	3	2.1%	
**Marital status**	Single	41	23.8%	2	1.4%	< 0.001
	Living in a partnership	129	75.0%	104	73.2%	
	Unknown	2	1.2%	36	25.4%	
**Relationship**	Partner			108	76.1%	
	Child			11	7.7%	
	Parent			18	12.7%	
	Close relative			5	3.5%	
**Education level**	**Low education level**					0.820
	No educational qualification	2	1.2%	2	1.4%	
	Basic school qualification	23	13.4%	22	15.5%	
	**Medium education level**					
	Secondary school certificate	21	12.2%	20	4.1%	
	Completed apprenticeship	68	39.5%	46	32.4%	
	**High education level**					
	High school graduation	15	8.7%	6	4.2%	
	Master craftsman’s diploma	12	7.0%	13	9.2%	
	University degree	28	16.3%	30	21.1%	
	Unknown	3	1.7%	3	2.1%	
**Work situation**	Full time	40	23.3%	60	42.3%	< 0.001
	Part time	23	13.4%	26	18.3%	
	Sick leave	15	8.7%	1	0.7%	
	Retired	75	43.6%	41	28.9%	
	Other	19	11.1%	14	9.8%	
**Diagnosis**	Glioblastoma	34	19.8%	–	–	–
	Astrocytoma	48	27.9%			
	Oligoastrocytoma	31	18.0%			
	/Oligodendroglioma					
	Meningioma	19	11.0%			
	Other	40	23.%			
**Tumour grading**	WHO I/II (“low grade”)	66	38.4%	–	–	–
	WHO III (“intermediate”)	52	30.2%			
	WHO IV (“high grade”)	54	31.4%			

Of the participating patients, 92 (43.5%) were in first-line treatment and 80 patients (46.5%) were in relapse. Regarding their tumors, 38.4% of patients had WHO grade I or II, 30.2% grade III, and 31.4% had grade IV tumors. Of all patients, 75.0% (*n* = 129) were living in a partnership at the time of the survey and 76.1% (*n* = 108) of the involved relatives were partners or spouses. Three (0.7%) patients received assistance from relatives or medical staff in completing the questionnaires due to difficulties with the German language.

### Access to psychooncologic care

At the Regensburg Brain Tumor Centre, 43.0% of patients (*n* = 74) and 30.3% of relatives (*n* = 43; *p* = 0.021) had had contact with psychooncology care during an out- or in-patient visit to the hospital. Offers of referral to psychooncology care were accepted by a comparable number of young and elderly patients (*p* = 0.728), with a contact rate of 41.9% among patients aged ≤35 years years (*n* = 13), 46.7% for 36–50 years (*n* = 21), 44.1% for 51–65 years (*n* = 26), and 56.0% among patients > 65 years of age (*n* = 14). With increasing tumor grade, contact to the psychooncology care showed a trend toward increasing (*p* = 0.054), from 36.1% in WHO grade I/II (*n* = 22) to 46.0% in WHO grade III (*n* = 23) and 59.2% in WHO grade IV (*n* = 29). Contact with psychooncologists did not vary significantly (*p* = 0.827) with education level (low: 43.5%, *n* = 10; middle: 49.4%, *n* = 41; high: 45.1%, *n* = 23).

### Psychooncologic need

Psychooncologic need was high among patients and relatives according to the HSI. More than half of the respondents (53.0% of patients, *n* = 85; and 58.2% of relatives, *n* = 78; *p* = 0.382) had a psychooncologic need (Additional file 2a) and 47.1% of patients (*n* = 81) answered that someone in their family is affected by the illness.

In comparison to their pre-illness state, 9.3% of patients (*n* = 16) and 19.7% of relatives (*n* = 28) had fewer social contacts to talk to about their fears and worries during the disease course (*p* = 0.047, Additional file 2b). Significantly less time to rest during the day was reported by 22.1% of patients (*n* = 38) and 35.9% of relatives (*n* = 51; *p* = 0.016).

Comparing mean HSI scores, patients’ psychooncologic need was dependent on age group, with lower need in the group aged ≤35 years (mean HSI score: 3.10, Additional file 3) and a significant peak in the 51–65 years age group (mean HSI score: 4.58, *p* = 0.027). Psychooncologic need was higher in patients with highly malignant tumors (mean HSI score: 4.40). Patients with low education levels tended to have a higher psychooncologic need (*n* = 24, mean HSI score: 4.58) when compared to patients with a high education level (*n* = 54, mean HSI score: 3.83; *p* = 0.534).

Patients had a significantly higher psychooncologic need (HSI: 4+ vs. 0–3, Additional file 4) while receiving chemotherapy (yes 68.2% vs. no 56.0%) than in phases without treatment (yes 20.0% vs. no 13.3%; *p* = 0.03). Patients felt significantly less adequately informed about their diagnosis (83.1 vs. 95.6%, *p* = 0.011) and treatment (81.7 vs. 94.4%, *p* = 0.016) if the psychooncologic need was high. The most significant uneven distribution was observed for depression: among patients with a high psychooncologic need, the rate of moderately or severely depressed patients was 58.8% compared to 5.3% in the group without psychooncologic need (*p* < 0.001). No significant relationship was seen with other factors such as sex, age, marital status, education level, working situation, WHO grade, tumor status, and time from diagnosis.

**Fig. 1:**
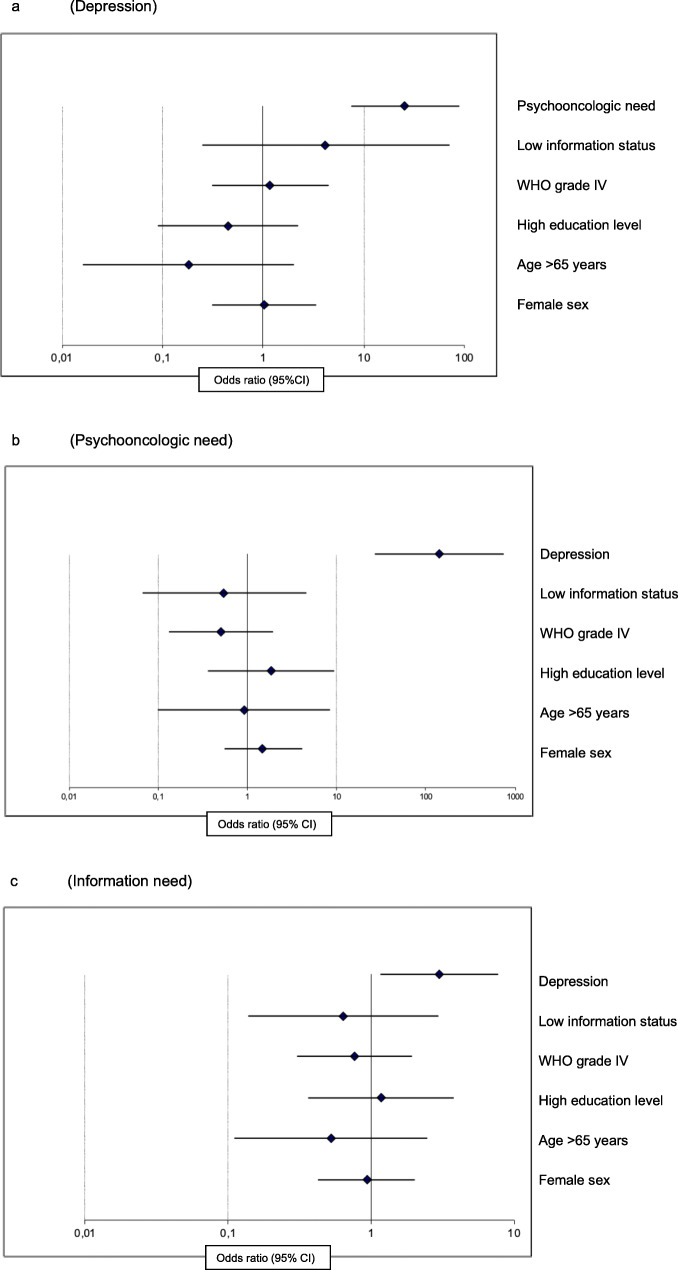
Impact of patient characteristics on depression, psychooncologic need, and information need. **a**: Multivariable binary logistic regression analysis of depression, showing the odds ratios of mild to severe vs no to minimal depression in relation to psychooncologic need, low information status, high WHO grade, high education level, higher age and female sex. A mild to severe depression relates significantly to a higher psychooncologic need. **b**: Multivariable binary logistic regression analysis of psychooncologic need, showing the odds ratios of psychooncologic need in relation to mild to severe depression, low information status, high WHO grade, high education level, higher age and female sex . A higher psychooncologic need relates significantly to a mild to sever depression. **c**: Multivariable binary logistic regression analysis of information need, showing the odds ratios of information need in relation to mild to severe depression, low information status, high WHO grade, high education level, higher age and female sex. Higher information need relates significantly to a mild to severe depression

In the univariable and multivariable regression analyses, psychooncologic need was not significantly related to any of the categories investigated except for depression levels. Here, patients with mild (OR 7.077, 95% CI 2.263–22.137; *p* = 0.001) and moderate to severe (OR 149.27; 95% CI 26.690–737.20; *p* < 0.001) depression levels had significantly higher psychooncologic need as opposed to patients with no to minimal depression (multivariable regression, Table 2; Fig. 1a).

**Table 2:** Multivariable regression analysis of patients’ psychooncologic need. Results of univariable and multivariable binary logistic regression analysis on psychooncologic need (Hornheider score 4+ vs 0–3; *N* = 160, missings not shown; significant differences are shown in bold)

		Univariable				Multivariable			
		*p*-value	odds ratio	95% CI		*p*-value	odds ratio	95% CI	
				lower	higher			lower	higher
Sex	Male		1.000				1.000		
	Female	0.317	1.379	0.735	2.587	0.422	1.501	0.556	4.051
Age	≤35		1.000				1.000		
	36–50	0.391	1.514	0.587	3.906	0.927	0.937	0.232	3.791
	51–65	0.172	1.840	0.768	4.413	0.771	0.803	0.183	3.513
	> 65	0.626	1.308	0.445	3.842	0.943	0.922	0.101	8.399
Marital status	Single		1.000				1.000		
	Partnership	0.989	0.995	0.479	2.066	0.511	0.676	0.210	2.171
Education level	Low		1.000						
	Middle	0.616	0.789	0.314	1.986	0.928	0.932	0.200	4.334
	High	0.497	0.714	0.270	1.886	0.460	1.844	0.363	9.356
Working situation	Full time		1.000				1.000		
	Part time	0.380	1.600	0.561	4.567	0.985	1.017	0.176	5.890
	Sick leave	0.402	1.676	0.501	5.611	0.532	0.539	0.078	3.737
	Retired	0.102	1.972	0.874	4.449	0.758	1.280	0.266	6.171
WHO grade	WHO I/II		1.000				1.000		
	WHO III	0.686	0.853	0.394	1.844	0.837	0.888	0.288	2.740
	WHO IV	0.936	1.031	0.495	2.146	0.311	0.503	0.133	1.899
Tumor status	Primary diagnosis		1.000				1.000		
	Relapse	0.827	1.072	0.575	1.998	0.384	0.607	0.197	1.869
Time from diagnosis/relapse (years)	< 1.0		1.000				1.000		
	1.0–4.9	0.339	0.690	0.322	1.478	0.485	0.611	0.153	2.438
	5.0+	0.560	0.762	0.306	1.900	0.172	0.344	0.074	1.592
	ns	0.188	0.500	0.178	1.405	0.121	0.304	0.067	1.369
Treatment status	Chemotherapy		1.000				1.000		
	Radiotherapy/surgery	0.342	0.294	0.024	3.671	0.690	0.251	0.000	221.94
	Follow-up	0.642	0.812	0.338	1.951	0.796	1.234	0.250	6.092
	No treatment	0.014	0.252	0.084	0.761	0.123	0.243	0.040	1.468
Information level (general)	Informed		1.000				1.000		
	Not informed	0.067	4.318	0.901	20.678	0.576	0.549	0.067	4.496
Depression (PHQ-9 score)	No/minimal (0–4)		1.000				1.000		
	Mild (5–9)	**< 0.001**	**5.000**	**2.035**	**12.282**	**0.001**	**7.077**	**2.263**	**22.137**
	Moderate/severe (10+)	**< 0.001**	**62.500**	**18.001**	**217.00**	**< 0.001**	**140.27**	**26.690**	**737.20**

### Depression burden

The depression burden was high in our analysis (Additional file 5). The result of the summed score analysis showed mild to severe depressive syndrome in 65.0% of patients (*n* = 112, mean score 10.6) and 66.9% of caregivers (*n* = 95, mean score 10.9). Clinically relevant scores > 10 were observed for 31.9% (*n* = 55) of patients and 28.9% (*n* = 41) of patients’ caregivers. In the categorical assessment, 15.7% of patients (*n* = 27) and 19.0% of relatives (*n* = 27) screened positive for major depression, with mean scores of 17.4 and 18.3, respectively. Suicidal thoughts on several days were declared by 28 patients and 17 relatives, including three in each group who experienced these thoughts on more than half of the days and four patients and six caregivers who experienced these thoughts nearly every day. If patients had consented to being contacted in these cases, they were advised to visit a psychiatrist or psychooncologist.

Mean depression levels significantly increased with age (Additional file 6). Patients ≤35 years (*n* = 28) had the lowest depression levels (mean = 6.27) when compared to patients aged 51–65 (*n* = 64, mean = 9.28, *p* = 0.017) or > 65 years (*n* = 25, mean = 9.12, *p* = 0.060). Although patients with WHO grade I–II (low-grade) tumors (*n* = 57) declared no diagnosis or treatment burden, the mean depression level (mean = 8.47) tended to be higher than in the patient group with WHO grade III tumors (*n* = 47, mean = 7.09; *p* > 0.05). Patients with WHO grade IV (high-grade) tumors (*n* = 51) had a significantly higher mean depression level (mean = 9.57; *p* = 0.024) compared to WHO grade III.

The patient group with a low education level (*n* = 21) had significantly higher summed depression scores (mean = 11.48) when compared to patients with a medium (*n* = 84, mean = 8.18; *p* = 0.012) or high education level (*n* = 47, mean = 7.38; *p* = 0.004).

The mean depression level was independent of disease status, namely first diagnosis or progression (mean = 8.6, *n* = 81 at first diagnosis vs. mean = 8.3, *n* = 74 with progressive disease; *p* = 0.711).

Both the distribution of patients’ characteristics according to depression (Additional file 7) and the results of univariable and multivariable logistic regression analyses show dependencies comparable to the relationships seen for psychooncologic need (Table 3, Fig. 1b): no significant dependency on sex, age, marital status, education level, working situation, WHO grade, tumor status, or time from diagnosis was observed.

**Table 3:** Results of univariable and multivariable binary logistic regression analysis on depression, yes vs. no according to PHQ-9 score (depression yes or no. 5+ vs. 0–4; *N* = 160, missings not shown; significant differences are shown in bold)

		Univariable				Multivariable			
		*p*-value	odds ratio	95% CI		*p*-value	odds ratio	95% CI	
				lower	higher			lower	higher
Sex	Male		1.000				1.000		
	Female	0.787	1.091	0.580	2.051	0.963	1.028	0.316	3.347
Age	≤35		1.000				1.000		
	36–50	0.298	1.647	0.643	4.218	0.089	3.530	0.824	15.129
	51–65	0.129	1.976	0.821	4.760	0.727	1.304	0.294	5.779
	> 65	0.649	1.273	0.451	3.590	0.163	0.181	0.016	2.003
Marital status	Single		1.000				1.000		
	Partnership	0.843	1.077	0.518	2.238	0.571	1.446	0.404	5.175
Education level	Low		1.000				1.000		
	Middle	0.873	0.925	0.359	2.389	0.801	1.214	0.269	5.473
	High	0.594	0.762	0.280	2.073	0.320	0.449	0.092	2.181
Working situation	Full time		1.000				1.000		
	Part time	0.406	1.556	0.549	4.409	0.443	2.347	0.266	20.743
	Sick leave	0.128	2.750	0.748	10.105	0.096	4.717	0.759	29.298
	Retired	0.009	2.947	1.312	6.621	0.097	0.158	0.018	1.394
WHO grade	WHO I/II		1.000				1.000		
	WHO III	0.881	1.059	0.499	2.247	0.620	0.724	0.201	2.601
	WHO IV	0.344	1.448	0.672	3.119	0.815	1.171	0.312	4.401
Tumor status	Primary diagnosis		1.000				1.000		
	Relapse	0.117	1.667	0.880	3.158	0.567	1.416	0.430	4.664
Time from diagnosis/relapse (years)	< 1.0		1.000				1.000		
	1.0–4.9	0.465	0.750	0.346	1.624	0.584	1.494	0.355	6.289
	5.0+	0.754	1.168	0.442	3.086	0.109	3.759	0.745	18.977
	ns	0.315	0.594	0.215	1.639	0.887	1.123	0.226	5.586
Treatment status	Chemotherapy		1.000				1.000		
	Radiotherapy/surgery	0.093	0.109	0.008	1.446	0.099	0.060	0.002	1.690
	Follow-up	0.132	0.447	0.157	1.275	0.115	0.238	0.040	1.418
	No treatment	**0.006**	**0.193**	**0.059**	**0.628**	0.344	0.394	0.057	2.715
Information level (diagnosis)	Informed		1.000				1.000		
	Not informed	**0.028**	**9.978**	**1.289**	**77.259**	0.316	4.200	0.254	69.436
Information level (treatment)	Informed		1.000				1.000		
	Not informed	**0.040**	**3.775**	**1.063**	**13.405**	0.756	1.416	0.158	12.666
Psychooncologic need	No (0–3)		1.000				1.000		
	Yes (4+)	**< 0.001**	**12.667**	**5.517**	**29.082**	**< 0.001**	**25.581**	**7.501**	**87.236**

The rate of treated patients was significantly higher among those with mild to severe depression (score of 5+ derived from PHQ-9) compared to patients with no to minimal depression (score 0–4, *p* = 0.018; Additional file 7). The number of patients who felt sufficiently informed about diagnosis and treatment was significantly lower among patients with mild or moderate to severe depression (diagnosis: 85.3 vs. 98.3%, *p* = 0.008; treatment: 83.2 vs. 94.9%, *p* = 0.029) than in patients with no or minimal depression. The proportion of patients with psychooncologic need was with 71.7% significantly higher among patients with mild to severe depression as opposed to 16.7% in patients with no or minimal depression (*p* < 0.001).

The observed relationships were confirmed by univariable logistic binary regression. Only the psychooncologic need continued to be significantly related to depression after risk adjustment by means of multivariable regression (Table 3, Fig. 1b), which corresponds to the results from regression on psychooncologic need.

### Information need

#### Comparison of patients and relatives

In our analysis, 41.5% of family caregivers (*n* = 59) did not feel sufficiently informed about the disease and its treatment, which was comparable to the information level in patients (38.4%, *n* = 66; *p* = 0.602).

More than half of the interviewed patients (59.9%; *n* = 103) and a significantly higher proportion of family caregivers (72.5%; *n* = 103; *p* = 0.019) wished to be fully informed about the cancer disease. The remaining participants wished to receive partial information in different extent. At the time of the survey, 63.4% (*n* = 109) of patients and 66.2% of relatives (*n* = 94; *p* = 0.354) had sought and obtained information on cancer-specific topics, with a clear preference for information on treatment and diagnosis. Concerning psychologic distress, 15.7% of patients (*n* = 27) and 16.2% (*n* = 23; *p* = 0.904) of family caregivers aimed to be informed on referral to psychooncology care (21.5%: *n* = 37) and sought support for psychological stress caused by the diagnosis (30.3%; *n* = 43; *p* = 0.070). In addition, 2.1% (*n* = 3) of the family caregivers had looked for information helplines.

The majority of patients (87.8%; *n* = 151) and family caregivers (78.2%; *n* = 111) felt adequately informed about their diagnosis and 84.3% (*n* = 145) of patients and 78.2% (*n* = 111) of relatives felt adequately informed about treatment. The number of family caregivers who did not feel adequately informed about the diagnosis or treatment of their sick family member was considerable, at 19.7 (*n* = 28) and 20.4% (*n* = 29), respectively. Patients who had a greater need for information tended to have a more distinctive information-seeking behavior than patients who preferred to leave the decisions to their physicians (*p* = 0.057).

#### Patients’ information need

From the distribution of patient characteristics depicted in Additional file 8 and the univariable and multivariable regression analysis (Table 4, Fig. 1c), only tendencies toward relationships to levels of patients’ information need can be derived.

**Table 4:** Multivariable regression analysis of patient information need. Results of univariable and multivariable binary logistic regression analysis on patients’ information need (*N* = 172, missings not shown; significant differences are shown in bold)

		Univariable				Multivariable			
		*p*-value	odds ratio	95% CI		*p*-value	odds ratio	95% CI	
				lower	higher			lower	higher
Sex	Male		1.000				1.000		
	Female	0.827	1.070	0.582	1.966	0.850	0.929	0.432	1.997
Age	≤35		1.000				1.000		
	36–50	0.374	0.659	0.263	1.653	0.336	0.601	0.213	1.696
	51–65	0.394	0.690	0.294	1.620	0.577	0.740	0.257	2.131
	> 65	0.022	0.275	0.090	0.833	0.409	0.521	0.111	2.447
Marital status	Single		1.000				1.000		
	In partnership	0.505	1.276	0.623	2.614	0.530	1.314	0.561	3.077
Education level	Low		1.000						
	Middle	0.809	1.118	0.453	2.759	0.706	1.230	0.421	3.595
	High	0.545	1.345	0.515	3.510	0.786	1.175	0.367	3.768
Working situation	Full time		1.000				1.000		
	Part time	0.758	0.850	0.303	2.386	0.655	0.749	0.211	2.661
	Sick leave	0.210	2.211	0.640	7.639	0.617	1.459	0.331	6.433
	Retired	0.181	0.586	0.268	1.282	0.265	0.505	0.152	1.680
WHO grade	WHO I/II		1.000				1.000		
	WHO III	0.540	1.257	0.605	2.610	0.697	1.186	0.504	2.789
	WHO IV	0.852	0.933	0.450	1.936	0.570	0.765	0.303	1.928
Tumor status	Primary diagnosis		1.000				1.000		
	Relapse	0.116	1.629	0.887	2.990	0.470	1.347	0.601	3.022
Time from diagnosis/relapse (years)	< 1.0		1.000				1.000		
	1.0–4.9	0.182	0.605	0.290	1.264	0.617	0.771	0.279	2.132
	5.0+	0.071	0.430	0.172	1.074	0.147	0.413	0.125	1.363
	ns	0.113	0.438	0.158	1.215	0.339	0.554	0.165	1.860
Treatment status	Chemotherapy		1.000				1.000		
	Radiotherapy/surgery	0.752	1.500	0.121	18.540	0.749	1.575	0.098	25.401
	Follow-up	0.076	0.466	0.200	1.083	0.538	0.684	0.204	2.291
	No treatment	0.430	0.667	0.244	1.825	0.629	1.403	0.356	5.524
Information level (general)	Informed		1.000				1.000		
	Not informed	0.923	1.063	0.311	3.629	0.567	0.639	0.138	2.958
Depression (PHQ-9 score)	No/minimal (0–4)		1.000				1.000		
	Mild (5–9)	0.243	1.558	0.740	3.277	0.113	2.004	0.848	4.734
	Moderate/severe (10+)	0.086	1.926	0.911	4.073	**0.022**	**3.007**	**1.175**	**7.695**

The only significant correlation was an increase in information need with increasing grades of depression not for mild (OR 2.004, 95% CI 0.848–4.734; *p* = 0.113), but for moderate to severe (OR 3.007, 95% CI 1.175–7.695; *p* = 0.022) depression vs. no or minimal depression. Furthermore, a non-significant decrease of information need with younger age, fewer therapies, and time from diagnosis or relapse was observed. Similarly, the small increase in information need with higher education level and living in a partnership did not reach significance. No dependency on the status of sufficient information was observed.

## Discussion

This study evaluated information need, psychooncologic need, need for referral to psychooncology care and depression in a large cohort of brain tumor patients and their family caregivers in conjunction with a number of potential confounding factors. The primary aim was to evaluate correlations between psychooncologic need, information need, and depression among patients with brain tumors and their family caregivers.

Patients with brain tumors are of specific interest, as they often suffer from neurocognitive problems and depression, that may influence psychooncologic and information need. In several publications, the need for referral to psychooncology care seems to be higher in patients with primary brain tumors compared to other cancer entities, with values ranging from 25 to 43% [16, 17, 29].

We also found that the majority of patients with primary brain tumors are in need for referral to psychooncology care. The extent of psychooncologic need correlates with distress, but not with age, sex, Karnofsky performance status (KPS), extend of resection, and current chemotherapy [7], which is in line with our data. In multivariable analysis, a significant positive correlation between psychooncologic need and depression as well as between depression and information need was detected.

Persons in the ≤35 years age group declared no need for referral to psychooncology care. This result may be misleading, as this younger sub-cohort may have a higher portion of lower-grade tumors (grade I/II), which are more prevalent at younger ages [30] and may include long-term survivors of childhood brain tumors. However, patients with low-grade tumors, patients aged 51–65 years and patients with a low education level all had less contact with the psychooncologic service teams, comparatively higher levels of depression, and a particularly high need for referral to psychooncology care, raising the hypothesis that access to certain aspects of care is lower in specific patient groups.

In our cross-sectional analysis, we did not find any correlation between disease status and psychooncologic need of the affected patients. However, other publications have shown that the need for referral to psychooncology care is particularly high in close temporal proximity to the time of disease progression or in situations that seem threatening for patients and caregivers [31]. This discordance could be explained by differences between study populations evaluated or by methodologic weaknesses of a cross-sectional investigation.

The psychologic burden borne by family caregivers appears to be particularly high, as relatives have even less psychosocial and social support than patients [32, 33]. Specifically, family caregivers struggle with how to adapt to cognitive changes in the patient and how to manage difficult aspects of the patient’s behavior and changing personality, both of which increase over time [9, 10]. This is reflected by a high and mostly unmet need for referral to psychooncology care for family caregivers in our analysis. Higher values for unmet needs are associated with higher distress levels in cross-sectional analysis [34].

More than a third of the respondents in the current study had a clinically relevant major depression. Other authors have described slightly lower rates for several tumor entities [35]. Therefore, depression in patients with primary brain tumors seems to be above the expected average of all cancer patients. Family caregivers usually rate the level of depression higher than the patients themselves, and are also more reliable than patients in reporting objective behavioral symptoms of depression [36]. Therefore, future studies should not only use self-assessment inventories to evaluate depression, but also family caregiver inventories.

Information need was high and partially unfulfilled in our study population. As also observed in similar studies, brain cancer patients often feel underinformed in early stages of their disease [8]. These observations indicate that communication of diagnosis, treatment, and prognosis should be adapted to patients’ personal needs [37].

Multivariable corrections were used in the present study to minimize possible confounding by known brain tumor-related prognostic factors [38] as well as additional tumor- and patient-specific factors that may influence psychooncologic need and depression. The correlation of psychooncologic need with information need and of depression with information need remained statistically significant in multivariable analysis. Therefore, the need for referral to psychooncology care is likely enhanced by a high rate of depression.

Several limitations of this work deserve mention. The survey was performed as a single-center study and at a specific cut-off day, implying that the time point was chosen independently of the specific follow-up status of patients. The patient population was therefore mixed, including both newly diagnosed and relapsed patients, and chronologic sequences could thus only be evaluated in a cross-sectional manner. Furthermore, regarding causality, it is unclear whether higher depression scores induce psychooncologic need or whether unmet psychooncologic need leads to depression. It is also unclear whether a high and unmet information need may induce psychooncologic need and depression. Additional research is needed to explore these aspects further.

## Conclusions

Our results imply that referral to psychooncology care represents a supportive therapy requirement in patients with brain tumors. We show that patients’ psychooncologic need and depression burden correlate strongly with counselling and information need. Considering these results, we hypothesize that adequate and repeated information regarding treatment as well as thorough psychooncologic screening and treatment of depression in patients and relatives alike are mandatory for adequate supportive therapy. Indeed, as also shown by others, referral to psychooncology care, sufficient time for face-to-face-meetings, and repeated surveys for patients and relatives are highly relevant for the therapeutic environment. Therefore, we propose that feasible and cost-effective supportive structures should be implemented for the patients and family caregivers who are most in need of them.

## Supplementary information

**Additional file 1.** CONSORT diagram. Five hundred twenty-two patients entered the study, of these 78 were diseased at the timepoint of the acquisition of the questionnaires. Therefore, 444 patients and their relatives were approached; 50.7% of these responded, with a ratio of 35.4% of the total population with a valid informed consent.

**Additional file 2.** Distribution of psychooncologic need of patients and family caregivers. A2a: Relative distribution of patients and relatives over the individual scores of the HSI. A2b: Differences in psychooncologic need in between patients and caregivers. Caregivers have significantly higher unmet need in two of the domains in comparison to patients. Psychoooncologic need was derived from Hornheider Screening Instrument, HIS.

**Additional file 3.** Patients’ need for referral to psychooncology care in relation to patient age, WHO grade and education level. A3a: Patients’ need for referral to psychooncology care depending on age. A3b: Patients’ need for referral to psychooncology care depending on diagnosis. A3c: Patients’ need for referral to psychooncology care depending on education level. The mean score for all items was derived from Hornheider Screening Instrument, HIS.

**Additional file 4. **Patient characteristics according to psychooncologic need. Absolute and relative distributions of demographic factors, tumor-related factors, information levels and depression score according to psychooncologic need yes vs no derived from Hornheider Screening Instrument, HSI. *N* = 160 HSI scores were collected; missing scores were excluded from the analysis; significant levels are shown in bold.

**Additional file 5.** Depression level of patients and family caregivers. Depression was evaluated with the PHQ-9 instrument and is depicted as absolute numbers of patients and caregivers in groups for no, minimal, mild, moderate and severe depression. The portion of mild to severe depressed patients or caregivers did not significantly differ between patients (65.0%) and caregivers (66.9%).

**Additional file 6.** Depression level in relation to age, WHO grade and education level. Depression was evaluated with the PHQ-9 instrument. A6a: Mean PHQ-9 score for depression depending on age. A6b: Mean PHQ-9 score for depression depending on WHO grade. A6c: Mean PHQ-9 score for depression depending on education level. The depression level is depicted as mean PHQ-9 score for all items.

**Additional file 7. **Patient characteristics according to depression level. Absolute and relative distributions of demographic factors, tumor-related factors, information levels and psychooncologic need in relation to depression level 5+ vs. 0–4 derived form PHQ-9 score are shown. *N* = 160 PHQ-9 scores were collected; missing scores were excluded from the analysis; significant levels are shown in bold.

**Additional file 8. **Patient characteristics according to information need. Absolute and relative distributions of demographic factors, tumor-related factors, information levels and depression level in relation to current information need are shown. Information need is depicted as informed vs. not informed; *N* = 172 information need questionnaires were collected; missing scores were excluded from the analysis; significant levels are shown in bold.

**Additional file 9.** Patient questionnaire.

**Additional file 10.** Caregivers questionnaire.

## Data Availability

This non-interventional investigation was not registered in a publicity accessible database. The datasets used during the current study are available from the corresponding author on request.

## Abbreviations

CNS: Central Nervous System
CI: Confidence Interval
HSI: Hornheider Screening Instrument
KPS: Karnofsky Performance Status
OR: Odd’s Ratio
PHQ: Patient Health Questionnaire
WHO: World Health Organisation
