# Supplementary material for: Single-institution cross-sectional study to evaluate need for information and need for referral to psychooncology care in association with depression in brain tumor patients and their family caregivers
Source: BMC Psychol. 2020 Sep 10;8:96. doi: 10.1186/s40359-020-00460-y (PMC7488319; doi:10.1186/s40359-020-00460-y)
Supplement: Supplementary file 1 — Additional file 1. CONSORT diagram. Five hundred twenty-two patients entered the study, of these 78 were diseased at the timepoint of the acquisition of the questionnaires. Therefore, 444 patients and their relatives were approached; 50.7% of these responded, with a ratio of 35.4% of the total population with a valid informed consent. [file 40359_2020_460_MOESM1_ESM.docx]

**A1: Consort diagram**

No difference between patients with low grade or high grade tumors in need for psychooncological support in the first 5 years after diagnosis or in the situation of progression

**
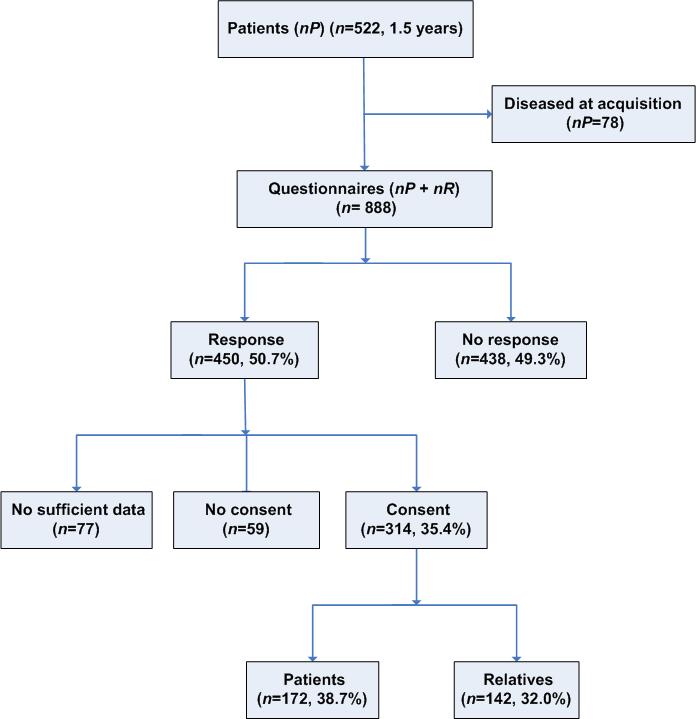
**

..
