# Supplementary material for: Single-institution cross-sectional study to evaluate need for information and need for referral to psychooncology care in association with depression in brain tumor patients and their family caregivers
Source: BMC Psychol. 2020 Sep 10;8:96. doi: 10.1186/s40359-020-00460-y (PMC7488319; doi:10.1186/s40359-020-00460-y)
Supplement: Supplementary file 2 — Additional file 2. Distribution of psychooncologic need of patients and family caregivers. A2a: Relative distribution of patients and relatives over the individual scores of the HSI. A2b: Differences in psychooncologic need in between patients and caregivers. Caregivers have significantly higher unmet need in two of the domains in comparison to patients. Psychoooncologic need was derived from Hornheider Screening Instrument, HIS. [file 40359_2020_460_MOESM2_ESM.docx]

**A2:** **Distribution of psychooncologic need of patients and family caregivers**

Figure A2a (relative distribution of patients and relatives)

53.0% Patients 58.2% Relatives with a total score $>4$

Figure A2b (differences in between patients and caregivers)
