# Supplementary material for: Single-institution cross-sectional study to evaluate need for information and need for referral to psychooncology care in association with depression in brain tumor patients and their family caregivers
Source: BMC Psychol. 2020 Sep 10;8:96. doi: 10.1186/s40359-020-00460-y (PMC7488319; doi:10.1186/s40359-020-00460-y)
Supplement: Supplementary file 3 — Additional file 3. Patients’ need for referral to psychooncology care in relation to patient age, WHO grade and education level. A3a: Patients’ need for referral to psychooncology care depending on age. A3b: Patients’ need for referral to psychooncology care depending on diagnosis. A3c: Patients’ need for referral to psychooncology care depending on education level. The mean score for all items was derived from Hornheider Screening Instrument, HIS. [file 40359_2020_460_MOESM3_ESM.docx]

**A3:** **Patients’ need for referral to psychooncology care in relation to patient age, WHO grade and education level**
Figure A3a (patient age)

mean HSI score

up to 35 years

above 65 years

51 to 65 years

36 to 50 years

p=0.027

Figure A3b (WHO grade)

mean HSI score

WHO grade I/II WHO grade III WHO grade IV

Figure A3c (education level)

mean HSI score
