# Supplementary material for: Single-institution cross-sectional study to evaluate need for information and need for referral to psychooncology care in association with depression in brain tumor patients and their family caregivers
Source: BMC Psychol. 2020 Sep 10;8:96. doi: 10.1186/s40359-020-00460-y (PMC7488319; doi:10.1186/s40359-020-00460-y)
Supplement: Supplementary file 4 — Additional file 4. Patient characteristics according to psychooncologic need. Absolute and relative distributions of demographic factors, tumor-related factors, information levels and depression score according to psychooncologic need yes vs no derived from Hornheider Screening Instrument, HSI. N = 160 HSI scores were collected; missing scores were excluded from the analysis; significant levels are shown in bold. [file 40359_2020_460_MOESM4_ESM.docx]

**A4: Patient characteristics according to psychooncologic needs**

|  | | Psychooncologic needs | | | | | |  |
| --- | --- | --- | --- | --- | --- | --- | --- | --- |
|  |  | No (0–3) | | Yes (4+) | | Total | | Chi^2^ |
|  |  | N | Column N % | N | Column N % | N | Column N % | *p*-value |
| Sex | Male | 35 | 46.7% | 33 | 38.8% | 68 | 42.5% | 0.317 |
|  | Female | 40 | 53.3% | 52 | 61.2% | 92 | 57.5% |  |
| Age | ≤35 | 17 | 22.7% | 13 | 15.3% | 30 | 18.8% |  |
|  | 36–50 | 19 | 25.3% | 22 | 25.9% | 41 | 25.6% | 0.573 |
|  | 51–65 | 27 | 36.0% | 38 | 44.7% | 65 | 40.6% |  |
|  | >65 | 12 | 16.0% | 12 | 14.1% | 24 | 15.0% |  |
| Marital status | Single | 18 | 24.0% | 20 | 24.1% | 38 | 24.1% | 0.989 |
|  | Partnership | 57 | 76.0% | 63 | 75.9% | 120 | 75.9% |  |
| Education level | Low | 10 | 13.3% | 14 | 16.9% | 24 | 15.2% |  |
|  | Middle | 38 | 50.7% | 42 | 50.6% | 80 | 50.6% | 0.793 |
|  | High | 27 | 36.0% | 27 | 32.5% | 54 | 34.2% |  |
| Working situation | Full time | 22 | 31.9% | 15 | 20.3% | 37 | 25.9% |  |
|  | Part time | 11 | 15.9% | 12 | 16.2% | 23 | 16.1% | 0.435 |
|  | Sick leave | 7 | 10.1% | 8 | 10.8% | 15 | 10.5% |  |
|  | Retired | 29 | 42.0% | 39 | 52.7% | 68 | 47.6% |  |
| WHO grade | WHO I/II | 29 | 38.7% | 34 | 40.0% | 63 | 39.4% |  |
|  | WHO III | 22 | 29.3% | 22 | 25.9% | 44 | 27.5% | 0.885 |
|  | WHO IV | 24 | 32.0% | 29 | 34.1% | 53 | 33.1% |  |
| Tumor status | Primary diagnosis | 41 | 54.7% | 45 | 52.9% | 86 | 53.8% |  |
|  | Relapse | 34 | 45.3% | 40 | 47.1% | 74 | 46.3% | 0.827 |
| Time from diagnosis/relapse (years) | <1.0 | 20 | 26.7% | 30 | 35.3% | 50 | 31.3% |  |
|  | 1.0–4.9 | 29 | 38.7% | 30 | 35.3% | 59 | 36.9% | 0.580 |
|  | 5.0+ | 14 | 18.7% | 16 | 18.8% | 30 | 18.8% |  |
|  | ns | 12 | 16.0% | 9 | 10.6% | 21 | 13.1% |  |
| Treatment status | Chemotherapy | 10 | 13.3% | 17 | 20.0% | 27 | 16.9% |  |
|  | Radiotherapy/surgery | 2 | 2.7% | 1 | 1.2% | 3 | 1.9% | **0.030** |
|  | Follow-up | 42 | 56.0% | 58 | 68.2% | 100 | 62.5% |  |
|  | No treatment | 21 | 28.0% | 9 | 10.6% | 30 | 18.8% |  |
| Information level (diagnosis) | Informed | 70 | 95.9% | 69 | 83.1% | 139 | 89.1% | **0.011** |
|  | Not informed | 3 | 4.1% | 14 | 16.9% | 17 | 10.9% |  |
| Information level (treatment) | Informed | 68 | 94.4% | 67 | 81.7% | 135 | 87.7% | **0.016** |
|  | Not informed | 4 | 5.6% | 15 | 18.3% | 19 | 12.3% |  |
| Information level (general) | Informed | 71 | 97.3% | 74 | 89.2% | 145 | 92.9% | **0.049** |
|  | Not informed | 2 | 2.7% | 9 | 10.8% | 11 | 7.1% |  |
| Depression (PHQ-9 score) | No/minimal (0–4) | 45 | 60.0% | 9 | 10.6% | 54 | 33.8% |  |
|  | Mild (5–9) | 26 | 34.7% | 26 | 30.6% | 52 | 32.5% | **<0.001** |
|  | Moderate/severe (10+) | 4 | 5.3% | 50 | 58.8% | 54 | 33.8% |  |
|  | Total | 75 | 100.0% | 85 | 100.0% | 160 | 100.0% |  |
