# Supplementary material for: Single-institution cross-sectional study to evaluate need for information and need for referral to psychooncology care in association with depression in brain tumor patients and their family caregivers
Source: BMC Psychol. 2020 Sep 10;8:96. doi: 10.1186/s40359-020-00460-y (PMC7488319; doi:10.1186/s40359-020-00460-y)
Supplement: Supplementary file 5 — Additional file 5. Depression level of patients and family caregivers. Depression was evaluated with the PHQ-9 instrument and is depicted as absolute numbers of patients and caregivers in groups for no, minimal, mild, moderate and severe depression. The portion of mild to severe depressed patients or caregivers did not significantly differ between patients (65.0%) and caregivers (66.9%). [file 40359_2020_460_MOESM5_ESM.docx]

**A5: Depression level of patients and family caregivers**

No difference between patients with low grade or high grade tumors in need for psychooncological support in the first 5 years after diagnosis or in the situation of progression

65.0% Patients 66.9% Relatives
