# Supplementary material for: Single-institution cross-sectional study to evaluate need for information and need for referral to psychooncology care in association with depression in brain tumor patients and their family caregivers
Source: BMC Psychol. 2020 Sep 10;8:96. doi: 10.1186/s40359-020-00460-y (PMC7488319; doi:10.1186/s40359-020-00460-y)
Supplement: Supplementary file 6 — Additional file 6. Depression level in relation to age, WHO grade and education level. Depression was evaluated with the PHQ-9 instrument. A6a: Mean PHQ-9 score for depression depending on age. A6b: Mean PHQ-9 score for depression depending on WHO grade. A6c: Mean PHQ-9 score for depression depending on education level. The depression level is depicted as mean PHQ-9 score for all items. [file 40359_2020_460_MOESM6_ESM.docx]

**A6: Depression level in relation to age, WHO grade and education level**

A6a (age)

mean PHQ-9 score

up to 35 years

36 to 50 years

51 to 65 years

above 65 years

*p*=0.060

*p*=0.017

A6b (WHO grade)

mean PHQ-9 score

*p*=0.024

A6c (depression level)

mean PHQ-9-score

*p*=0.012

*p*=0.004
