# Supplementary material for: Single-institution cross-sectional study to evaluate need for information and need for referral to psychooncology care in association with depression in brain tumor patients and their family caregivers
Source: BMC Psychol. 2020 Sep 10;8:96. doi: 10.1186/s40359-020-00460-y (PMC7488319; doi:10.1186/s40359-020-00460-y)
Supplement: Supplementary file 7 — Additional file 7. Patient characteristics according to depression level. Absolute and relative distributions of demographic factors, tumor-related factors, information levels and psychooncologic need in relation to depression level 5+ vs. 0–4 derived form PHQ-9 score are shown. N = 160 PHQ-9 scores were collected; missing scores were excluded from the analysis; significant levels are shown in bold. [file 40359_2020_460_MOESM7_ESM.docx]

**A7**: **Patient characteristics according to depression level**

|  | | Depression PHQ-9 score | | | | | |  |
| --- | --- | --- | --- | --- | --- | --- | --- | --- |
|  |  | 0–4 | | 5+ | | Total | | Chi^2^ |
|  |  | *N* | Column *N* % | *N* | Column  *N %* | *N* | Column *N* % | *p*-value |
| Sex | Male | 27 | 45.0% | 48 | 42.9% | 75 | 43.6% | 0.787 |
|  | Female | 33 | 55.0% | 64 | 57.1% | 97 | 56.4% |  |
| Age | ≤35 | 14 | 23.3% | 17 | 15.2% | 31 | 18.0% |  |
|  | 36–50 | 15 | 25.0% | 30 | 26.8% | 45 | 26.2% | 0.453 |
|  | 51–65 | 20 | 33.3% | 48 | 42.9% | 68 | 39.5% |  |
|  | >65 | 11 | 18.3% | 17 | 15.2% | 28 | 16.3% |  |
| Marital status | Single | 15 | 25.0% | 26 | 23.6% | 41 | 24.1% | 0.843 |
|  | Partnership | 45 | 75.0% | 84 | 76.4% | 129 | 75.9% |  |
| Education level | Low | 8 | 13.6% | 17 | 15.5% | 25 | 14.8% |  |
|  | Middle | 30 | 50.8% | 59 | 53.6% | 89 | 52.7% | 0.815 |
|  | High | 21 | 35.6% | 34 | 30.9% | 55 | 32.5% |  |
| Working situation | Full time | 20 | 38.5% | 20 | 19.8% | 40 | 26.1% |  |
|  | Part time | 9 | 17.3% | 14 | 13.9% | 23 | 15.0% | 0.053 |
|  | Sick leave | 4 | 7.7% | 11 | 10.9% | 15 | 9.8% |  |
|  | Retired | 19 | 36.5% | 56 | 55.4% | 75 | 49.0% |  |
| WHO grade | WHO I/II | 25 | 41.7% | 41 | 36.6% | 66 | 38.4% |  |
|  | WHO III | 19 | 31.7% | 33 | 29.5% | 52 | 30.2% | 0.613 |
|  | WHO IV | 16 | 26.7% | 38 | 33.9% | 54 | 31.4% |  |
| Tumor status | Primary diagnosis | 37 | 61.7% | 55 | 49.1% | 92 | 53.5% | 0.116 |
|  | Relapse | 23 | 38.3% | 57 | 50.9% | 80 | 46.5% |  |
| Time from diagnosis/relapse (years) | <1.0 | 16 | 26.7% | 35 | 31.3% | 51 | 29.7% |  |
|  | 1.0–4,9 | 25 | 41.7% | 41 | 36.6% | 66 | 38.4% | 0.587 |
|  | 5.0+ | 9 | 15.0% | 23 | 20.5% | 32 | 18.6% |  |
|  | ns | 10 | 16.7% | 13 | 11.6% | 23 | 13.4% |  |
| Treatment status | Chemotherapy | 5 | 8.3% | 23 | 20.5% | 28 | 16.3% |  |
|  | Radiotherapy/surgery | 2 | 3.3% | 1 | 0.9% | 3 | 1.7% | **0.018** |
|  | Follow-up | 35 | 58.3% | 72 | 64.3% | 107 | 62.2% |  |
|  | No treatment | 18 | 30.0% | 16 | 14.3% | 34 | 19.8% |  |
| Information level (diagnosis) | Informed | 58 | 98.3% | 93 | 85.3% | 151 | 89.9% |  |
|  | Not informed | 1 | 1.7% | 16 | 14.7% | 17 | 10.1% | **0.008** |
| Information level (treatment) | Informed | 56 | 94.9% | 89 | 83.2% | 145 | 87.3% |  |
|  | Not informed | 3 | 5.1% | 18 | 16.8% | 21 | 12.7% | **0.029** |
| Information level (general) | Informed | 59 | 100.0% | 98 | 89.9% | 157 | 93.5% | **0.012** |
|  | Not informed | 0 | 0.0% | 11 | 10.1% | 11 | 6.5% |  |
| Psychooncologic need | No (0–3) | 45 | 83.3% | 30 | 28.3% | 75 | 46.9% | **<0.001** |
|  | Yes (4+) | 9 | 16.7% | 76 | 71.7% | 85 | 53.1% |  |
|  | Total | 54 | 100.0% | 106 | 100.0% | 160 | 100.0% |  |
