# Supplementary material for: Single-institution cross-sectional study to evaluate need for information and need for referral to psychooncology care in association with depression in brain tumor patients and their family caregivers
Source: BMC Psychol. 2020 Sep 10;8:96. doi: 10.1186/s40359-020-00460-y (PMC7488319; doi:10.1186/s40359-020-00460-y)
Supplement: Supplementary file 8 — Additional file 8. Patient characteristics according to information need. Absolute and relative distributions of demographic factors, tumor-related factors, information levels and depression level in relation to current information need are shown. Information need is depicted as informed vs. not informed; N = 172 information need questionnaires were collected; missing scores were excluded from the analysis; significant levels are shown in bold. [file 40359_2020_460_MOESM8_ESM.docx]

**A8:** **Patient characteristics according to information need**

|  | | Current information need | | | | | |  |
| --- | --- | --- | --- | --- | --- | --- | --- | --- |
|  |  | Yes | | No | | Total | | Chi^2^ |
|  |  | *N* | Column *N* % | *N* | Column *N* % | *N* | Column *N* % | *p*-value |
| Sex | Male | 32 | 42.7% | 43 | 44.3% | 75 | 43.6% | 0.827 |
|  | Female | 43 | 57.3% | 54 | 55.7% | 97 | 56.4% |  |
| Age | ≤35 | 17 | 22.7% | 14 | 14.4% | 31 | 18.0% |  |
|  | 36–50 | 20 | 26.7% | 25 | 25.8% | 45 | 26.2% |  |
|  | 51–65 | 31 | 41.3% | 37 | 38.1% | 68 | 39.5% | 0.130 |
|  | >65 | 7 | 9.3% | 21 | 21.6% | 28 | 16.3% |  |
| Marital status | Single | 16 | 21.6% | 25 | 26.0% | 41 | 24.1% | 0.504 |
|  | Partnership | 58 | 78.4% | 71 | 74.0% | 129 | 75.9% |  |
| Education level | Low | 10 | 13.5% | 15 | 15.8% | 25 | 14.8% |  |
|  | Middle | 38 | 51.4% | 51 | 53.7% | 89 | 52.7% | 0.794 |
|  | High | 26 | 35.1% | 29 | 30.5% | 55 | 32.5% |  |
| Working situation | Full time | 19 | 48.7% | 21 | 53.8% | 40 | 51.3% |  |
|  | Part time | 10 | 25.6% | 13 | 33.3% | 23 | 29.5% | 0.117 |
|  | Sick leave | 10 | 25.6% | 5 | 12.8% | 15 | 19.2% |  |
|  | Retired | 26 | 40.0% | 49 | 55.7% | 75 | 49.0% |  |
| WHO grade | WHO I/II | 28 | 37.3% | 38 | 39.2% | 66 | 38.4% |  |
|  | WHO III | 25 | 33.3% | 27 | 27.8% | 52 | 30.2% | 0.726 |
|  | WHO IV | 22 | 29.3% | 32 | 33.0% | 54 | 31.4% |  |
| Tumor status | Primary diagnosis | 35 | 46.7% | 57 | 58.8% | 92 | 53.5% | 0.115 |
|  | Relapse | 40 | 53.3% | 40 | 41.2% | 80 | 46.5% |  |
| Time from diagnosis/relapse (years) | <1.0 | 28 | 37.3% | 23 | 23.7% | 51 | 29.7% |  |
|  | 1.0–4.9 | 28 | 37.3% | 38 | 39.2% | 66 | 38.4% | 0.210 |
|  | 5.0+ | 11 | 14.7% | 21 | 21.6% | 32 | 18.6% |  |
|  | ns | 8 | 10.7% | 15 | 15.5% | 23 | 13.4% |  |
| Treatment status | Chemotherapy | 16 | 21.3% | 12 | 12.4% | 28 | 16.3% |  |
|  | Radiotherapy/surgery | 2 | 2.7% | 1 | 1.0% | 3 | 1.7% | 0.249 |
|  | Follow-up | 41 | 54.7% | 66 | 68.0% | 107 | 62.2% |  |
|  | No treatment | 16 | 21.3% | 18 | 18.6% | 34 | 19.8% |  |
| Information level (diagnosis) | Informed | 65 | 87.8% | 86 | 91.5% | 151 | 89.9% | 0.436 |
|  | Not informed | 9 | 12.2% | 8 | 8.5% | 17 | 10.1% |  |
| Information level (treatment) | Informed | 61 | 84.7% | 84 | 89.4% | 145 | 87.3% | 0.373 |
|  | Not informed | 11 | 15.3% | 10 | 10.6% | 21 | 12.7% |  |
| Information level (general) | Informed | 69 | 93.2% | 88 | 93.6% | 157 | 93.5% | 0.923 |
|  | Not informed | 5 | 6.8% | 6 | 6.4% | 11 | 6.5% |  |
| Depression (PHQ-9 Score) | No/minimal (0–4) | 21 | 28.0% | 39 | 40.2% | 60 | 34.9% |  |
|  | Mild (5–9) | 26 | 34.7% | 31 | 32.0% | 57 | 33.1% | 0.213 |
|  | Moderate/severe (10+) | 28 | 37.3% | 27 | 27.8% | 55 | 32.0% |  |
|  | Total | 75 | 100.0% | 97 | 100.0% | 172 | 100.0% |  |
