# Supplementary material for: Single-institution cross-sectional study to evaluate need for information and need for referral to psychooncology care in association with depression in brain tumor patients and their family caregivers
Source: BMC Psychol. 2020 Sep 10;8:96. doi: 10.1186/s40359-020-00460-y (PMC7488319; doi:10.1186/s40359-020-00460-y)
Supplement: Supplementary file 9 — Additional file 9. Patient questionnaire. [file 40359_2020_460_MOESM9_ESM.docx]

**Patient survey**

Pilot study to determine information needs of patients with tumors of the central nervous system and their relatives.

Version 1.0 - as of: 14.08.2014

**Collection of medical / sociodemographic data**

| Age: | ________ years |  | | | |
| --- | --- | --- | --- | --- | --- |
| Gender: | □ female | □ male |  |  | |
| Marital status: | □ I live alone | □ I live in a permanent partnership | | | |
| Kids: | □ yes | □ no | | | |
| Nationality: | □ German | □ __________________________ | | | |
| Education level: | □ No degree  □ A-levels  □ University degree | □ Secondary school  □ Training | □ medium maturity  □ Master | | |
| Work situation: | □ Working □ Full time □ Part time  □ Housework | □ Sick leave  □ Unemployed | □ Pension  □ | | |
| Tumor diagnosis: | □ Astrocytoma  □ Oligoastrocytoma  □ Oligodendroglioma | □ Glioblastoma  □ Lymphoma  □ Other | □ Ependymoma □ Meningioma  ____________________ | | |
| □ I do not know the exact diagnosis | | | | | |
| WHO grade: | □ I □ II | □ III | □ IV | | |
| Date of initial diagnosis: ___ ___ ______  DD MM YYYY | | | | |  |

| Please mark with a cross what applies to you **currently** (multiple answers are possible)  □ I only recently learned of my diagnosis.  □ I just had surgery.  □ I am currently undergoing chemotherapy. □ I am currently undergoing radiation therapy.  □ I am participating in a therapy study. □ I am in post-treatment care.  □ No therapy is currently required. | | | | | |
| --- | --- | --- | --- | --- | --- |
| Current medical condition: | □ first diagnosis | □ relapse  □1. □2. □3. | | □ Currently no tumor detectable (remission) | |
| Date of last relapse: | | | |  | ___ ___ ______  DD MM YYYY |
|  | | |  |  | |
| My treatment in the neurology department at the University Hospital of Regensburg was ...  □ stationary | | | □ outpatient | □ both | |
| Did you have the opportunity to talk to the Psycho-Oncological Service during your stay? | | | □ yes | □ no | |
| Did you have the opportunity to talk to the social services during your stay? | | | □ yes | □ no | |
| About my diagnosis, I feel sufficiently informed. | | | □ yes | □ no | |
| About my current treatment, I feel sufficiently informed. | | | □ yes | □ no | |

**Please answer the following questions by ticking the number that best applies to you in the last week. There are no "right" or "wrong" answers.**

**[EORTC QLQ-C30] – NOT TRANSLATED**

**[EORTC QLQ-BN20] – NOT TRANSLATED**

**[PHQ 9] – NOT TRANSLATED**

**Survey of information needs**

**[in house-developed questionnaire]**

| 1. To what extent would you like to be informed in principle?  □ I want to know everything about □ Diagnosis □ Therapy □ Prognosis  □ Not more than absolutely necessary to □ Diagnosis □ Therapy □ Prognosis | | | | |
| --- | --- | --- | --- | --- |
| 2. To what extent do you want to be involved in treatment decisions?  □ I want to be involved in all decisions.  □ Depends on situation. □ I prefer to leave the necessary decisions to the doctors. | | | | |
| 3. How would you like to be informed? | | | | |
| □ In a personal conversation  □ Through individual advice by e-mail  □ Through brochures / flyers  □ Through CD / Video | | □ By telephone  □ In an internet forum  □ On information pages on the internet  □ Through mobile apps |  | |
| 4. Which additional counselling facilities do you know outside the University Hospital Regensburg? | | | | |
| □ German Cancer Aid e.V.  □ German Cancer Society  □ Tumor Center e.V. | □ Kid´s Cancer Information Service  □ Psychosocial cancer advice centre  □ _______________________________ | | |  |

| 5. What do you value in the information? | **Especially relevant** | **Relevant** | **Less relevant** |
| --- | --- | --- | --- |
| That everything is explained in **detail and comprehensively.** |  |  |  |
| That those who provide information or advice can be **trusted.** |  |  |  |
| That the information is **up-to-date**. |  |  |  |
| That an **easily understandable language** is used. |  |  |  |
| That the information is **individually** tailored to your questions and needs. |  |  |  |
| That the information provider/consultancy service provider is independent and **neutral**. |  |  |  |
| That there is the possibility of a **conversation in private**. |  |  |  |
| That the possibility of a conversation **with my relatives** exists. |  |  |  |
| That the information will help you to make **your own decision**. |  |  |  |
| That you can get **free** information and advice. |  |  |  |
| That the information is **scientifically sound**. |  |  |  |
| That you get a **quick** answer to a question. |  |  |  |
| That the information is **concise and precise**. |  |  |  |
| That you name **further contact persons** you can turn to. |  |  |  |
| That you know **what the information is based on**. |  |  |  |
| That the contact persons are **easy to reach**. |  |  |  |
| That you get all the important **information from one source.** |  |  |  |
| That you can get **information material to read**. |  |  |  |
| That the information is available around the **clock**. |  |  |  |

| 6. Have you already been informed / advised on tumour-specific topics? | | | | |
| --- | --- | --- | --- | --- |
|  | □ yes | □ no | | |
| 7. What was the reason for the search for information? | | | | |
| □ General information about the disease  □ Search for contact details / contact persons  □ Search for a specific counselling/decision support | | | | |
| 8. What information channels did you use for this? (multiple answers are possible) | | | | |
| □ Specialist  □ Internist  □ Oncologist  □ Neurologist □ Friends / acquaintances / relatives  □ Newspapers / magazines  □ Articles in professional journals  □ Television broadcasts  □ Lectures / Events  □ Information centres | | | □ Family doctor  □ Books / Guide / Information material  □ Internet forums  □ Information pages on the Internet  □ Persons concerned  □ Health Insurance |  |
| 9. On which DIAGNOSIS topics did you already inform yourself / have you been consulted (multiple answers are possible) | | | | |
| □ Symptoms / typical courses of disease  □ Expertise on the disease pattern  □ Current research results | | □ Chances of survival / prognosis □ Structure/function of the brain / neuroanatomy  □ Explanation / translation of technical terms | | |
| □ Medical examinations / test procedures (e.g. MRT, CT)  □ _____________________________________________ | | | | |
| 10. On which TREATMENT topics did you already inform yourself / have you been consulted (multiple answers are possible) | | | | |
| □ Suitable treatment facilities  □ Standard therapy  □ Consequences of non-treatment  □ Duration of treatment  □ Benefits / risks of treatment | | □ Quality of service providers  □ Surgery  □ Chemotherapy  □ Radiotherapy  □ Efficacy / Mode of action of the treatment  □ Further treatment options  □ Participation in clinical studies | | |
| □ Alternative treatment options as a supplement or alternative to conventional medicine  □ Incense □ Mistletoe □ Acupuncture □ Homeopathy □ Hyperthermia □ _________ | | | | |

| 11. On which topics in the area of LIFE WITH CANCER did you already inform yourself / have you been consulted (multiple answers are possible) | |
| --- | --- |
| □ Dealing with treatment consequences  □ Effects on everyday life  □ Maintenance / Improvement of Quality of Life  □ Maintenance of Independence  □ Sports | □ Dealing with side effects (prophylaxis)  □ Nutrition / food supplements  □ Sexuality / Desire for children  □ Travel  □ Cancer Follow-Up |
| □ Dealing with symptoms ( e.g: epilepsy / memory disorders / change of character speech disorders / paralysis/ etc. )  □ Dealing with the disease and the mental strain  □ Application / Intake Medication  □ Temodal □ CCNU □ Procarbazin □ Avastin □ Seizure medication □ Cortisone □ ______ _____ | |
| 12. On which topics in the area of FURTHER HELP / CONTACT ADDRESSES did you already inform yourself / have you been consulted (multiple answers are possible) | |
| □ Contact person in case of emergency  □ Psycho-oncological consultation  □ Patient interest groups  □ Organisation of the treatment (piloting)  □ Organization of rehabilitation / cure | □ Information for children  □ Hotline  □ Discussion group for relatives  □ Terminal care / palliative therapy / hospice  □ Active support / home care (medical care in the last phase of life) |
| 13. On which topics in the area of LEGAL QUESTIONS / ECONOMIC ASPECTS did you already inform yourself / have you been consulted (multiple answers are possible) | |
| □ Patient rights  □ Patient Decree / Power of Attorney  □ Last Will and Testament  □ Costs of the therapy  □ Severe disability | □ Privacy policy  □ Legal support  □ Services of the health insurance funds / Cost absorption  □ Care level / care allowance  □ Pension |

| 14. On which topics can we support you with current information?  □ DIAGNOSIS  ________________________________________________________________________________  ________________________________________________________________________________  ________________________________________________________________________________  □ TREATMENT  ________________________________________________________________________________  ________________________________________________________________________________  ________________________________________________________________________________  □ LIVING WITH CANCER  ________________________________________________________________________________  ________________________________________________________________________________  ________________________________________________________________________________  □ FURTHER ASSISTANCE / CONTACT ADDRESSES  ________________________________________________________________________________  ________________________________________________________________________________  ________________________________________________________________________________  □ LEGAL QUESTIONS / ECONOMIC AID  ________________________________________________________________________________  ________________________________________________________________________________  ________________________________________________________________________________ |
| --- |

**Finally, please answer the following questions about the current mental / psychological strain**

**[Hornheider Screening Instrument] – NOT TRANSLATED**

| If you would like to make a message or complaint about your current treatment in general, you have the opportunity to do so here:  ________________________________________________________________________________  ________________________________________________________________________________  ________________________________________________________________________________  ________________________________________________________________________________  ________________________________________________________________________________  ________________________________________________________________________________  ________________________________________________________________________________  ________________________________________________________________________________  ________________________________________________________________________________  **MANY THANKS FOR YOUR SUPPORT!** |
| --- |
